# Integrating Clinical and Multiple Omics Data for Prognostic Assessment across Human Cancers

**DOI:** 10.1038/s41598-017-17031-8

**Published:** 2017-12-05

**Authors:** Bin Zhu, Nan Song, Ronglai Shen, Arshi Arora, Mitchell J. Machiela, Lei Song, Maria Teresa Landi, Debashis Ghosh, Nilanjan Chatterjee, Veera Baladandayuthapani, Hongyu Zhao

**Affiliations:** 1 Division of Cancer Epidemiology and Genetics, National Cancer Institute, National Institute of Health, Bethesda, MD 20892 USA; 2 NSABP Foundation, Pittsburgh, PA 15212 USA; 3 Department of Epidemiology and Biostatistics, Memorial Sloan-Kettering Cancer Center, New York, NY 10021 USA; 4 Department of Biostatistics and Informatics, University of Colorado Denver, Aurora, CO 80045 USA; 5 Department of Biostatistics, Bloomberg School of Public Health, Johns Hopkins University, Baltimore, MD 21205 USA; 6 Department of Oncology, School of Medicine, Johns Hopkins University, Baltimore, MD 21205 USA; 7 Department of Biostatistics, The University of Texas M. D. Anderson Cancer Center, Houston, TX 77230 USA; 8 Department of Biostatistics, Yale School of Public Health, New Haven, CT 06520 USA

## Abstract

Multiple omic profiles have been generated for many cancer types; however, comprehensive assessment of their prognostic values across cancers is limited. We conducted a pan-cancer prognostic assessment and presented a multi-omic kernel machine learning method to systematically quantify the prognostic values of high-throughput genomic, epigenomic, and transcriptomic profiles individually, integratively, and in combination with clinical factors for 3,382 samples across 14 cancer types. We found that the prognostic performance varied substantially across cancer types. mRNA and miRNA expression profile frequently performed the best, followed by DNA methylation profile. Germline susceptibility variants displayed low prognostic performance consistently across cancer types. The integration of omic profiles with clinical variables can lead to substantially improved prognostic performance over the use of clinical variables alone in half of cancer types examined. Moreover, we showed that the kernel machine learning method consistently outperformed existing prognostic signatures, suggesting that including a large number of omic biomarkers may provide substantial improvement in prognostic assessment. Our study provides a comprehensive portrait of omic architecture for tumor prognosis across cancers, and highlights the prognostic value of genome-wide omic biomarker aggregation, which may facilitate refined prognostic assessment in the era of precision oncology.

## Introduction

Developing models that accurately predict patient survival using prognostic and predictive biomarkers is increasingly important in clinical research and practice^1,2^. Advances in high-throughput genomic technologies and large-scale sequencing studies including The Cancer Genome Atlas (TCGA) and the International Cancer Genome Consortium (ICGC) project have generated a rich resource of multi-dimensional omics data. Building cancer prognostic models incorporating genomic data has the potential to improve the precision of predicting patient clinical outcomes, to help understand the mechanism of tumor progression, and to evaluate the clinical values of biomarkers in clinical trials. Meanwhile, the complexity of the tumor genome poses a great challenge for cancer prognostic assessment. Indeed, substantial omic heterogeneity has been revealed for histologically homogeneous tumors in terms of genomics^3,4^, epigenomics^5^, transcriptomics^6–8^, and proteomics^9^. Recognizing its importance and challenges, the Cancer Moonshot Blue Ribbon Panel has recently recommended prediction of patient outcomes as one research area poised for acceleration^10^.

Considerable effort has been devoted to incorporating omic profiles into prognostic assessments for various cancer types. The earlier analyses typically created prognostic indices consisting of a few dozen selected genes based on microarray gene expression^11–13^. More recent works investigated multiple omic profiles for predicting survival in single cancer types^14,15^ and across few cancer types^16^. These studies focused on identifying a list of prognostic genes, molecules or signatures, excluded somatic mutations from analyses, and did not comprehensively consider the combination of multiple omic profiles. There is a need to conduct a pan-cancer analysis of prognostic accuracy for multiple omic profiles on a genome-wide scale, and to understand the shared patterns for prognostic performance of omic profiles across cancer types.

We hypothesized that prognosis-relevant signals may come from multiple pathways and involve a large number of omic biomarkers, the effect of which may be visible only when aggregated. Indeed, the omic biomarkers with moderate or weak prognostic value likely failed to reach the genome-wide prognostic significance threshold and consequently were discarded from the model. Therefore, the potential prognostic value of omic biomarkers may be unfulfilled and the underlying assumption of omic architecture of tumor prognosis built upon sparsity (a small subset of omic biomarkers driving prognosis) needs to be reconsidered. In such situations, a viable hypothesis is that a large number of omic biomarkers (with a continuum of effect size) are involved in prognosis. Hence, aggregating prognosis effects of all biomarkers (through a shrinkage model) may be more effective and stable, and provide improved assessments of prognostic performance of omic biomarkers.

**Figure 1:**
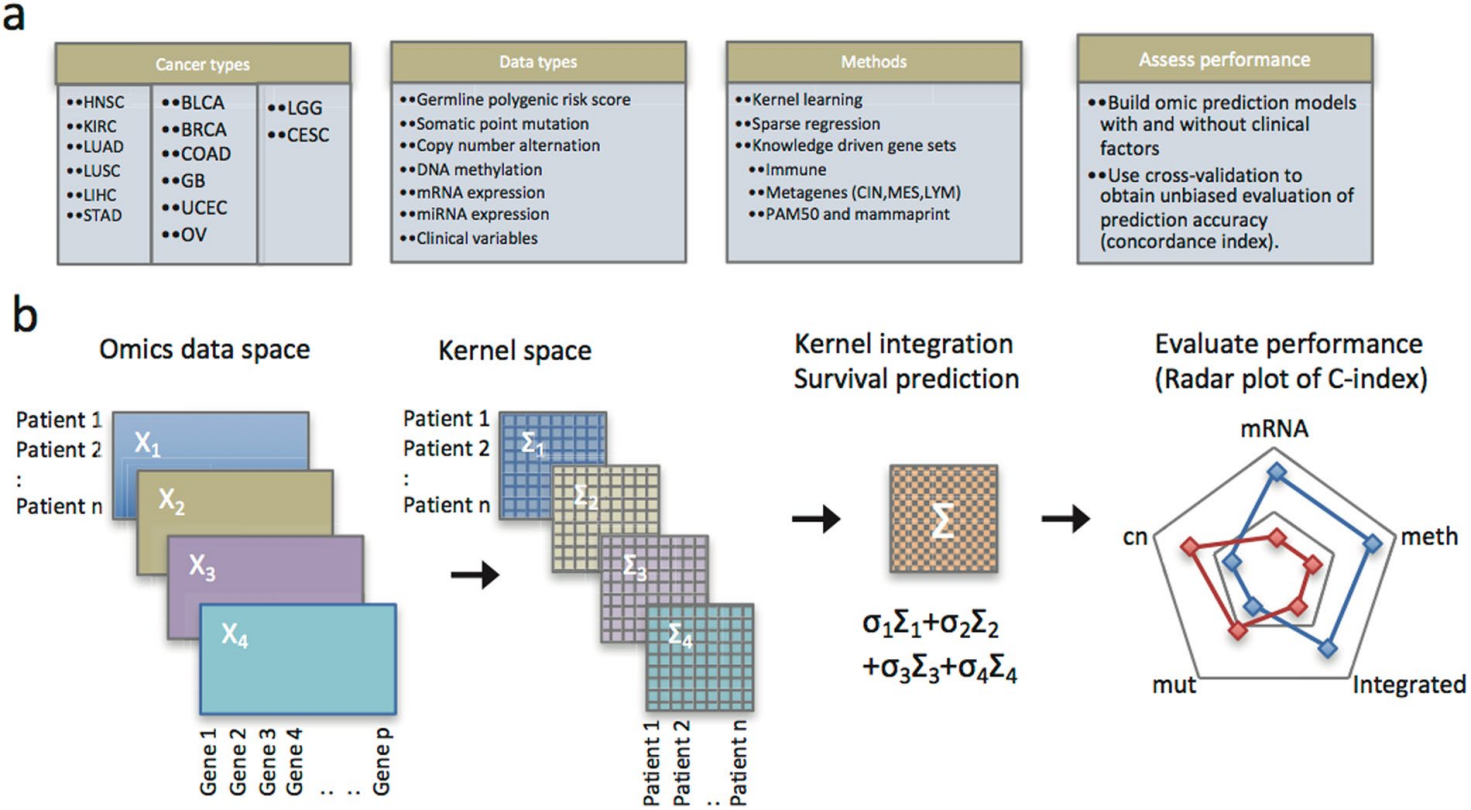
Overview of methods, cancer types, and omic data types. CIN: chromosomal instability; MES: mesenchymal transition; LYM: lymphocyte-specific immune recruitment.

To examine this hypothesis, we developed a multi-omic kernel machine learning method including all molecular markers of an omic profile simultaneously. The schematic analysis steps are illustrated in Fig. 1. We considered multiple omic profiles, including somatic mutations, DNA copy number, DNA methylation, mRNA expression, miRNA expression, and their combinations. In an effort to capture translational and post-translational regulations, functional protein analysis using reverse-phase protein arrays (RPPA) was added to the TCGA effort to integrate proteomic characterization of tumors with already available genomic, transcriptomic and clinical information^9^. Thus, we included analysis of the RPPA data set for prognostic assessment in the TCGA pan-cancer cohort.

The ability of each omic biomarker to predict overall survival was treated as a random variable and shrunken toward zero with a data-driven degree of shrinkage. In other words, omic architecture involving tumor prognosis was built upon all biomarkers, whose prognosis effects varied from very weak (for most biomarkers) to moderate (for some biomarkers) to strong (for a few biomarkers) in a continuous spectrum. This approach was not intended to identify individual prognosis-related biomarkers in a particular omic profile, which likely requires thousands of samples^17^, but to aggregate the prognostic effects of all biomarkers across various omic profiles and to quantify the prognostic value of tumor molecular profiles, alone or combined with clinical factors across cancer types. The basic idea behind the proposed multi-omic kernel machine learning method is intuitively simple: a patient’s predicted outcome would be similar to that of other patients with similar clinical variables and omic profiles, i.e. “someone like you”, measured by omic similarity matrices.

## Results

### Omic similarity matrices

**Figure 2:**
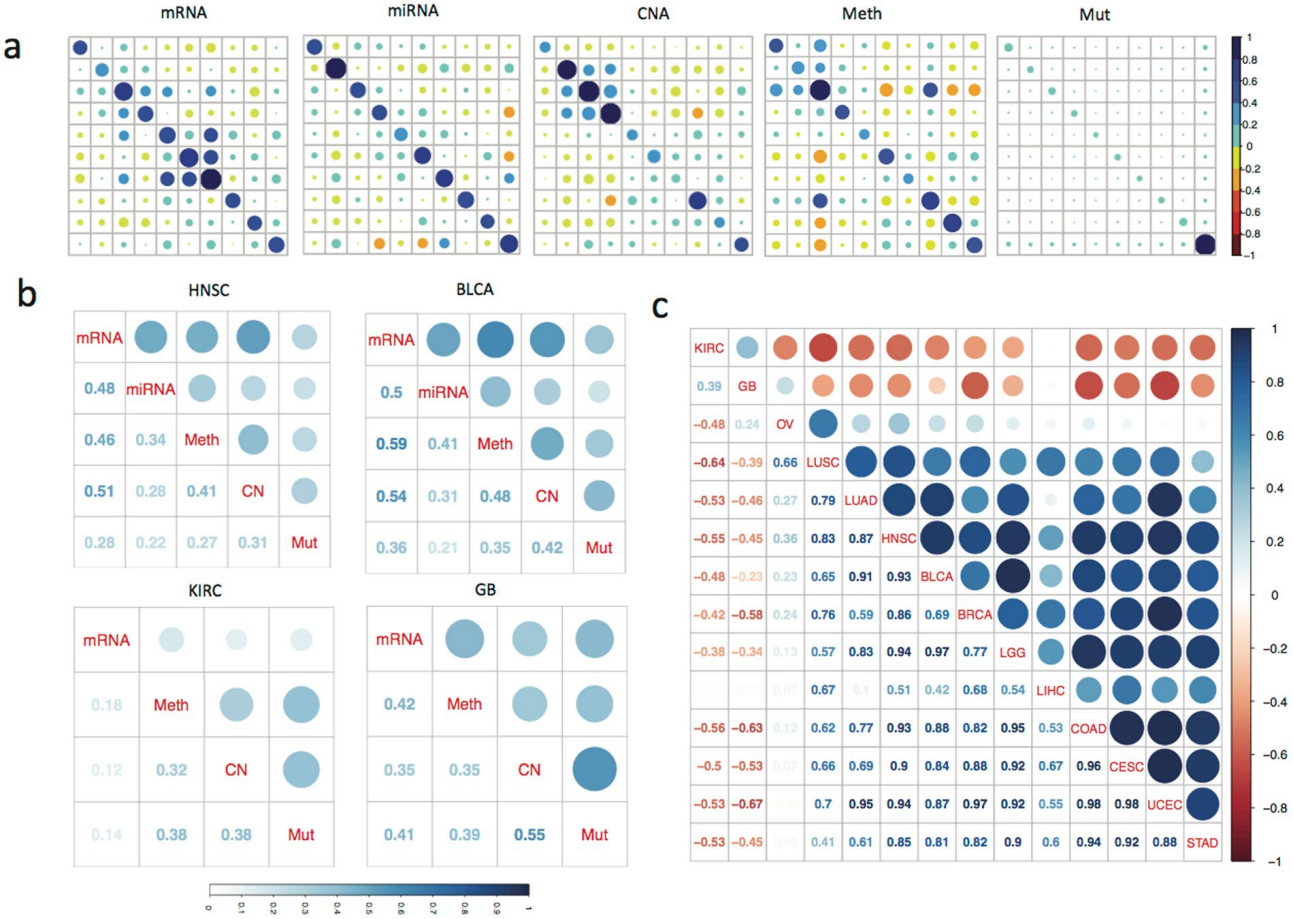
Omic similarity matrix and kernel alignment. (**a**) Illustration of the omic similarity matrix based on each individual data type including mRNA, miRNA, DNA methylation, copy number and somatic mutation in 10 randomly selected head and neck squamous cell carcinoma (HNSC) tumor samples. The diagonal elements in each matrix correspond to the average absolute value in the corresponding data type (scaled to be between 0 and 1) for a subject. For example, the dark blue circle in the lower right corner of the somatic mutation similarity matrix indicates high mutation burden for the corresponding patient sample compared to the others. The off-diagonal elements reflect the similarity between paired subjects. Each matrix was standardized to enhance contrast; (**b**) kernel alignment between different data types (averaged over all patient samples) in HNSC, BLCA, KIRC and GB as profile alignment matrices. They quantify the degree to which two subjects similar in one omic profile (e.g., gene expression) are also similar in another omic profile (e.g., methylation). Size of the circle and shade of the blue is proportional to the alignment value. Higher value indicates the better alignment between two omic profiles; (**c**) comparing profile alignment patterns across cancer types. Values close to one indicate similar alignment patterns between two cancer types.

For somatic mutation, DNA copy number, DNA methylation, mRNA and miRNA expression, an omic similarity matrix was computed for each omic data type using a linear kernel function that measures the similarity of omic profiles between subjects. Other kernel functions, including the Gaussian kernel, may be used, but at the cost of additional kernel parameters which may require a large sample for model tuning. We aggregated all biomarkers from each individual omic profile to create the corresponding omic similarity matrix (Methods). Fig. 2a illustrates examples of omic similarity patterns among ten head and neck squamous cell carcinoma (HNSC) patients based on their mRNA, miRNA, DNA methylation, copy number, and somatic mutation profiles, respectively. The diagonal elements reflect the average absolute level of all biomarkers for a subject. For example, the diagonals of the somatic mutation omic similarity matrix correspond to the normalized mutation burden for each subject. The off-diagonal elements evaluate the similarities and dissimilarities between paired subjects. The kernel regression method we propose leverages between-subject similarities for predicting patients’ survival outcome. We observed weak to moderate similarity in general between subjects for mRNA, miRNA, methylation and copy number profiles, while the somatic mutation profiles were almost unique with little similarity, if any, between subjects.

### Kernel alignment assessment

Next, we applied a kernel alignment approach^18^ to evaluate whether the omic similarity matrix defined by one omic profile (e.g., mRNA) aligned well with that defined by another (e.g., DNA methylation). The resulting profile alignment matrices measure the similarity between omic similarity matrices in each cancer type (Methods). Figure 2b shows the profile alignment matrices of HNSC, for which the highest alignments are between mRNA, DNA methylation and DNA copy number. In most of the cancer types we analyzed (Supplementary Figure 1), omic similarity matrices are positively aligned and mRNA aligns closely with miRNA, methylation and copy number, but weakly with somatic mutation. Interestingly, there exists a strong alignment between copy number and somatic mutation similarity matrices in kidney renal clear cell carcinoma (KIRC) and glioblastoma (GB). In stomach adenocarcinoma (STAD) and urothelial bladder carcinoma (BLCA), strong alignment was observed between mRNA and methylation similarity matrices.

To identify if there are cross-cancer type similarities in profile alignment matrices, we performed cancer type alignment as illustrated in Fig. 2c. Notably, the profile alignment matrix of KIRC is positively aligned with that of GB, largely due to their strong alignment between copy number and somatic mutation. Interesting, the profile alignment matrix of HNSC is most similar to the profile alignment matrix of lower grade glioma (LGG) and uterine corpus endometrial carcinoma (UCEC), followed by these of colon adenocarcinoma (COAD) and BLCA. In short, by generating omic similarity matrices, profile alignment matrices, and cancer type alignment matrix sequentially, we revealed the similarities between subjects for each omic profile, similarities between omic profiles in each cancer type, and similarities between cancer types in a hierarchical fashion. Omic similarity matrices will be used in the proposed multi-omic kernel machine learning method.

### Variation of prognostic performances across cancer types and their similarity within a cancer type

**Figure 3:**
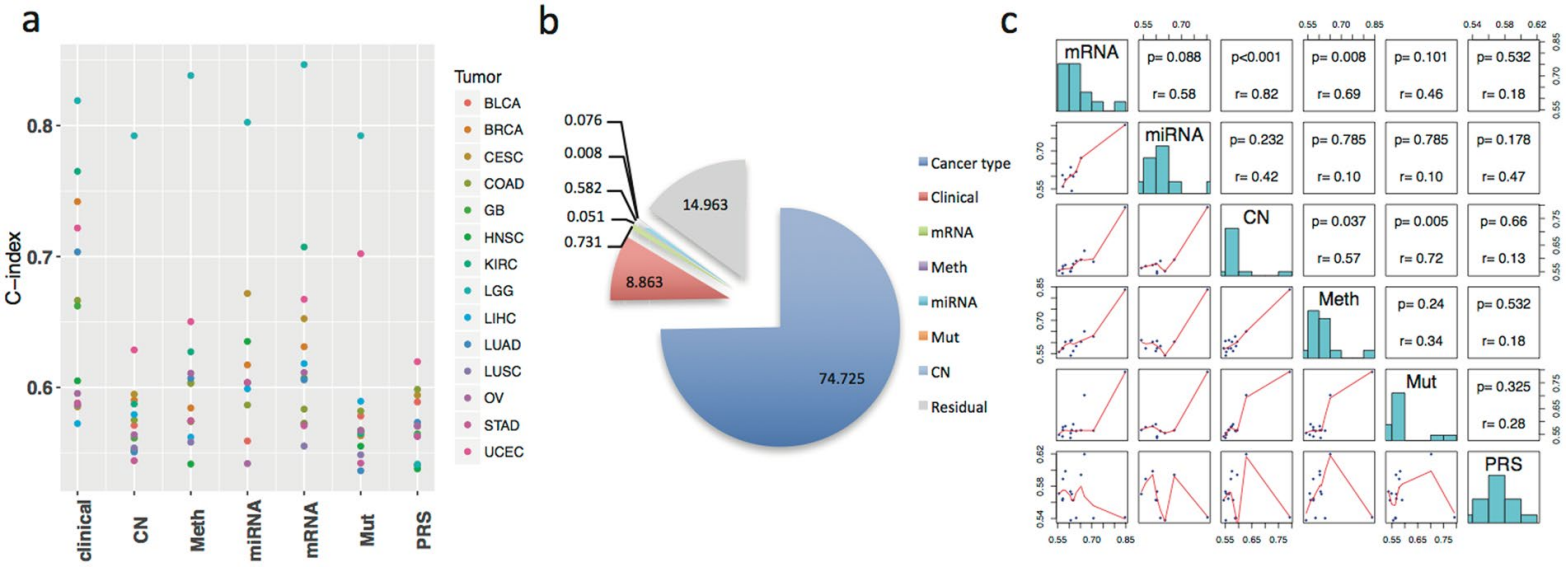
Patterns of variation in concordance index (C-index) across omic platforms and cancer types. (**a**) Scatterplot of the cross-validated C-index across cancer types and omic platforms; (**b**) proportions of variation explained by cancer type, clinical factors, and omic platforms respectively; (**c**) the pairwise Spearman correlation pattern in C-index between omic platforms.

We first applied the multi-omic kernel machine learning method to evaluate the prognostic performance of individual omic profile and clinical variables for 14 cancer types. The concordance index (C-index) was calculated to evaluate the concordance of the actual survival outcome and survival outcomes as predicted by either the kernel machine learning method based on the omic data or by a Cox proportional hazard model based on clinical variables. From a pan-cancer perspective, we observed that the prognostic power of clinical variables and some molecular profiles vary substantially across cancer types (Fig. 3a). For example, C-indices for clinical variables range from 0.572 in liver hepatocellular carcinoma (LIHC) (age, sex and tumor stage) to 0.819 in LGG (age, sex, tumor grade and histology) with standard deviation (SD) of 0.081. C-indices for mRNA range from 0.555 (LUSC) to 0.847 (LGG) with SD of 0.076. On the other hand, other omic profiles including DNA copy number (range: 0.544 (STAD) −0.792 (LGG) and SD: 0.063) and somatic point mutation (range: 0.536 (lung adenocarcinoma, LUAD) −0.792 (LGG) and SD: 0.071), show relatively less prognostic power and vary to a lesser degree between cancer types. Germline polygenic risk scores (PRS) had the weakest prognostic power in general; the C-indices in 13 of 14 cancer types are less than 0.6, leading to a small SD (0.025). This is not surprising since PRS consisting of common single nucleotide polymorphisms (SNPs) were identified in a genome-wide association study (GWAS)^19,20^ as being associated with cancer risk rather than prognosis.

The noticeable correlations of C-indices between molecular profiles and with clinical variables imply that the prognostic performance of molecular profiles and clinical variables largely depends on the cancer type, which is consistent with a previous report^16^. To systematically understand the variations in C-indices, we applied a linear mixed effects model to quantify the contribution of cancer type to the variation of the C-index for various omic profiles and clinical factors across cancer types, treating C-indices generated by individual molecular profiles and clinical variables as repeated prognosis evaluations for a given cancer type. The model suggested that the cancer type itself explained 74.7% of variation (see Methods) in prognostic performance while the remaining 25.3% of variation is due to clinical variables, molecular profiles, and other unidentified factors (Fig. 3b).

Within a given cancer type, the prognostic powers of molecular profiles are similar to each other, most noticeably for BLCA, LUSC and LGG (Fig. 3a) with some exceptions detailed later. Indeed, the correlation of C-indices between mRNA and copy number (spearman correlation rho = 0.824, Fig. 3c) across cancer types is most apparent, followed by copy number and somatic mutation (rho = 0.719), while the correlation of C-indices between miRNA and somatic mutation (rho = −0.103) is low. As for the group of clinical variables, its C-index is most highly correlated with C-indices of methylation and mRNA (rho = 0.648 and 0.495 respectively, Supplementary Figure 2). C-indices of germline PRS showed an inverse correlation with C-indices of miRNA (rho = −0.467) and weak correlations with other omic profiles. In subsequent sections, germline PRS was excluded because of its weak prognostic powers and correlations.

### Comparison of prognostic performance of omic profiles and clinical variables

**Figure 4:**
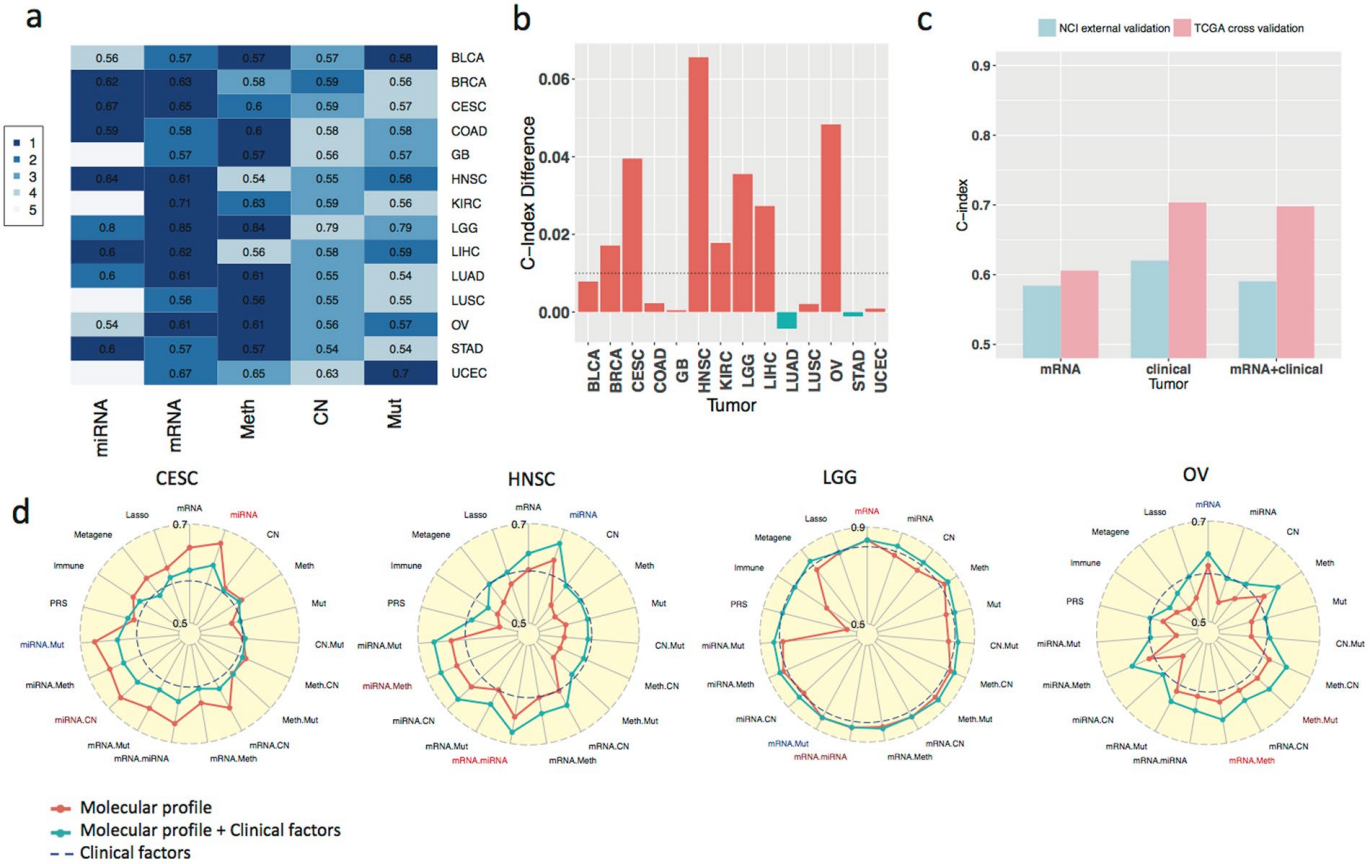
Prognostic performance of omic platforms across cancer types. (**a**) C-indices of individual omic platform for each cancer type (row). A C-index of 0.5 indicates random prediction, and values close to 1.0 correspond to the perfect prediction of survival outcome. The colors represent rankings of C-indices for a cancer type (the darker color corresponding the higher rank). (**b**) The most significant improvement in C-index by combining the omic profile with clinical variables among five omic profiles for each cancer type (e.g., an increase of 0.066 in C-index, representing 10.8% improvement, was observed in HNSC). Positive values indicate additional gains by combining molecular and clinical predictors over using clinical factors alone. (**c**) Validation of the lung adenocarcinoma mRNA kernel machine learning model developed in the TCGA data and validated in a National Cancer Institute (NCI) study cohort. (**d**) Radar plots of C-index for individual and combinations of omic platforms in four cancer types. Blue dotted line benchmarks the performance of the clinical variables.

We compared the individual molecular profiles with clinical variables in terms of prognostic performance measured by the C-index. Among the molecular profiles, mRNA, miRNA, and methylation frequently show the highest C-indices among all platforms (Fig. 4a). Although the prognostic powers of copy number and somatic mutations are relatively weak, combining them with other omic profiles can lead to increased prognostic power in particular cancer types (e.g. combining copy number with mRNA in KIRC, Supplementary Figure 3 radar plot).

Although the prognostic powers of omic profiles alone are on average weaker than the prognostic power of clinical variables by 0.051 (P = 1.05*10^−5^ based on Wald statistic; details in Methods), there were a number of notable exceptions in specific cancer types (Fig. 4d). In endocervical adenocarcinoma (CESC), mRNA and miRNA perform significantly better (C-indices: 0.653 and 0.672 respectively) than tumor stage and age (C-index: 0.585). In HNSC, miRNA (C-index: 0.635) outperforms clinical variables including age, stage and grade (C-index: 0.605). This may be due to the fact that HPV infection status was a missing data point for most HNSC cases and thus excluded from the analysis as a clinical variable. On the other hand, it suggests that incorporating omic profiles is able to complement the absence of known or unknown prognosis-related clinical factors for prognostic assessment. In LGG, mRNA and methylation (C-indices: 0.847 and 0.838) outperform clinical variables including age, gender, tumor grade and histological subtypes (astrocytoma, oligodendroglioma and oligo-astrocytoma histologies, C-index: 0.819). In ovarian serous cystadenocarcinoma (OV), C-indices for mRNA and methylation (0.611 and 0.611, respectively) are higher than that for age, stage, grade and residual disease (0.595). Finally, we observed better prognostic performance for mRNA in LIHC (C-index: 0.618) and miRNA in STAD (C-index: 0.604) than that of the corresponding clinical factors (C-indices: 0.572 and 0.588 respectively). Note that C-indices are very low for both clinical variables and omic profiles in LUSC and BLCA, reflecting the difficulty of prognostic prediction for these cancer types.

Figure 4a shows that mRNA and miRNA, followed by DNA methylation, are consistently the top performers across cancer types. To further quantify this rank, we explicitly modeled and compared the contribution of individual omic profiles to prognostic prediction across cancer types (Methods). This comparison showed that the C-index of mRNA is above the average prognostic power of omic profiles by 0.025 (P = 7.47*10^−8^), the best performance among the five omic profiles we analyzed. The second best omic profile is miRNA with a C-index above the average by 0.020 (P = 4.08*10^−5^), followed by DNA methylation with an elevated C-index above the average by 0.008 (P = 0.09). By contrast, the C-indices of copy number and somatic mutation are below the average prognostic power by 0.005 and 0.002 respectively. The differences, however, are not statistically significant (P = 0.219 and 0.618 respectively).

We next investigated whether combining multiple molecular profiles would further improve prognostic power in comparison with the individual molecular profiles. Although the molecular profile combination would lead to the strongest prognostic power in a few cancer types, such as HNSC and OV, the increment from the best individual omic profile is often minimal. Compared to the single molecular profiles, the combination of two molecular profiles would improve C-index by 0.008 (P = 0.04) on average. This may reflect the similarity between omic profiles both in terms of omic similarity matrices and C-indices by individual omic profiles.

### Prognostic powers of mRNA-based signatures

A number of mRNA-based prognostic signatures have been proposed and adapted for prognostic assessment. Prognostic signatures commonly consist of either pre-selected mRNA transcripts (based on previous studies capturing well-characterized biological processes such as immune infiltration^8^ and chromosomal instability^15^), or agnostically selected mRNA transcripts (determined using penalized Cox regression, random survival forests, or other statistical and machine learning methods^16^). The underlying assumption of these studies is that tumor prognosis is driven by a relatively small number of mRNA transcripts, commonly less than one hundred. As will be illustrated shortly, a much larger number of mRNA transcripts, likely thousands of them, each with a weak prognostic effect individually, may be involved in the tumor prognosis for highly heterogeneous tumor types. Therefore, we systematically compared various established signatures with the kernel machine learning method we developed that aggregates prognostic effect across all transcripts of annotated genes from the mRNA profile.

Specifically, we compared the prognostic powers of (a) the kernel machine learning method for genome-wide aggregation of mRNA transcripts; (b) pre-specified prognostic signatures, including the metagene signatures^15^ and the ESTIMATE immune signatures^6^ developed across multiple cancer types; (c) the PAM50 breast cancer classifier^21^ or the MammaPrint signature^22^ that predicts distant metastasis for early stage breast cancer; (d) LGG subtypes defined by IDH1 mutation and co-deletion of chromosome 1p/19q; and (e) algorithmically selecting mRNA transcripts by L1 penalized Cox regression (LASSO)^16^.

**Figure 5:**
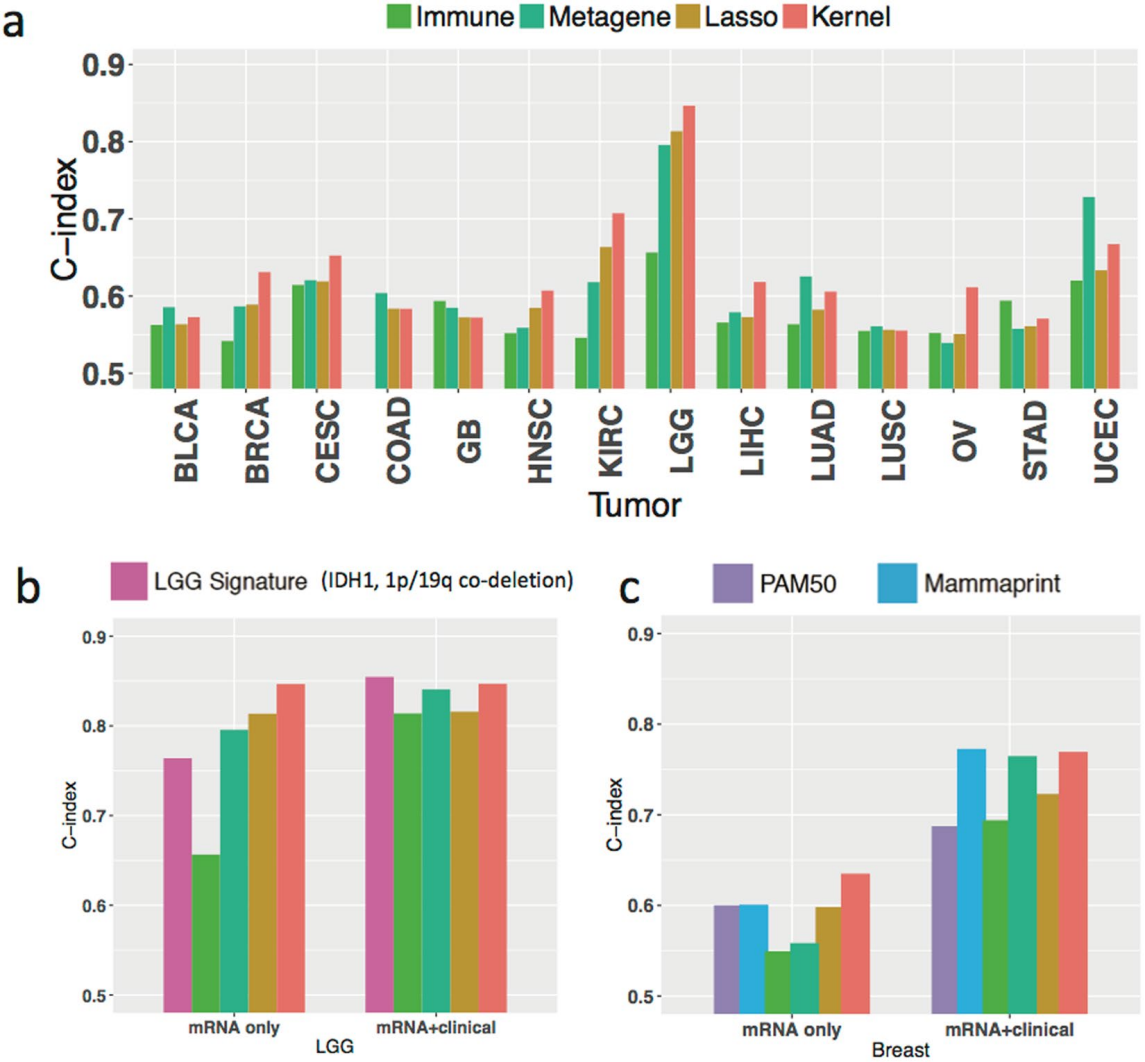
Comparing kernel prediction with the established prognostic signatures. (**a**) Bar plots of C-index for the kernel machine learning method, an immune infiltration signature, a Metagene signature (capturing chromosome instability, mesenchymal transition, and lymphocyte immune recruitment), and data-driven selection of mRNA transcripts using Lasso-regression across cancer types. (**b**) Extended analysis of low grade glioma (LGG) including the IDH1, chromosome 1p/19q co-deletion prognostic subtypes. (**c**) Extended analysis of BRCA including the PAM50 classification and the MammaPrint signature.

We found that the kernel machine learning method outperforms the metagene and immune signatures in 7 cancer types (Fig. 5a). On average, the kernel method improves C-index over the metagene and immune signatures by 0.018 and 0.052 (P = 0.14 and 1.62*10^−4^) respectively, across cancer types. For KIRC, the metagene and immune signatures lead to significantly lower C-indices (0.618 and 0.546) as compared to the kernel method (0.707). There are a few exceptions in which the metagene and immune signatures perform slightly better than the kernel method, including the metagene signature in UCEC (C-index = 0.728 v.s 0.667 by the kernel machine learning method), in LUAD (C-index = 0.626 v.s 0.606), in COAD (C-index = 0.604 v.s 0.583), and in GB (C-index = 0.585 v.s 0.572), as well as the immune signature in STAD (0.594 v.s 0.571) and in GB (C-index = 0.594 v.s 0.572). This implies that these two biological signatures may be relevant to the prognosis in particular cancer types but not universally.

In LGG, the metagene and lasso signatures achieve slightly higher C-indices than the LGG subtypes (Fig. 5b) and the kernel machine learning method achieves the highest C-index. In breast cancer (BRCA), the kernel machine learning method also outperforms the PAM50 subtype classification (luminal A, luminal B, HER2, Basal-like, Normal-like) and the MammaPrint signature (Fig. 5c).

The model including only agnostically selected mRNA transcripts by LASSO performs worse in prognostic prediction than the kernel machine learning method (on average by 0.025 across cancer types, P = 0.04) among 11 of 14 cancer types, most notably in OV (LASSO C-index = 0.551, vs 0.611 for the kernel machine learning method). In the remaining three cancer types (GB, LUSC and COAD), C-indices of agnostically selected mRNA transcripts by LASSO (C-index = 0.572, 0.556 and 0.584) and aggregated mRNA transcripts combined via the kernel machine learning method (C-index = 0.572, 0.555 and 0.583) are very close, suggesting that either the prognostic power of mRNA is generally weak or the number of prognosis-related mRNA transcripts is limited in these cancer types.

Taken together, these results suggest that traditional prognosis methods relying on a small number of molecular biomarkers, such as Lasso-derived genomic signatures, may not be sufficient to achieve the optimal prognostic performance in some cancer types. The kernel machine learning method provides more refined prognostic prediction by aggregating a large number of molecular features in the half of the cancer types we analyzed.

### Enhancing prognostic powers by integrating clinical variables with molecular profiles

Combining clinical variables with molecular profiles shows increased C-indices (up to 10.8% in HNSC for miRNA) compared to using clinical variables alone in 7 of 14 cancer types (by at least 0.01, Fig. 4b). In particular, integrating clinical variables with miRNA or mRNA profiles improves C-index by 0.012 and 0.010 respectively (P = 0.03 and 0.06) on average across cancer types, but this is not the case with methylation, copy number, and somatic point mutation (P = 0.50, 0.68, and 0.93 respectively). The improvement of prognostic power by integrating clinical variables with molecular profiles for CESC, HNSC, LIHC, LGG, and OV is not surprising, since the prognostic power of mRNA and/or miRNA is greater for those cancers than that of clinical variables alone; consequently, the integration of clinical variables with molecular profiles yields enhanced prognostic power over clinical variables alone, to various degrees. Notably, for HNSC and OV the prognostic power obtained by combining clinical variables with molecular profiles is stronger than that yielded using either molecular profiles or clinical variables alone (e.g. C-indices for clinical variables 0.605, mRNA 0.607, clinical variables + mRNA 0.640 in HNSC; C-indices for clinical variables 0.595, mRNA 0.611, clinical variables + mRNA 0.635 in OV). Although not so pronounced as in HNSC and OV, the improved prognostic prediction realized by integrating clinical variables and molecular profiles is also observed in BRCA and KIRC. In the remaining 7 cancer types, the combination of clinical variables and molecular profiles had similar prognostic power of clinical variables alone. For example, the incorporation of mRNA profile in LUAD did not improve the prognostic power of the clinical variables (age, gender, and tumor stage). We confirmed these results in the National Cancer Institute (NCI) whole-exome sequencing study of LUAD (Fig. 4c)^23^.

### Validation in NCI LUAD RNA-seq study

We further validated the proposed kernel machine learning method in the NCI RNA-seq study of 101 lung adenocarcinoma samples, which have been processed with the same bioinformatics pipelines as samples from TCGA. The details of sample collection and study population have been reported previously^23^. We used samples from the TCGA LUAD study as the training samples, applied the kernel machine learning method and validated the fitted model in the NCI LUAD study. This external validation resulted in a C-index of 0.584 for mRNA, slightly lower than the figure that cross-validation in TCGA LUAD study produced, 0.606 (3.63% reduction, Fig. 4c). In contrast, external validation of clinical variables resulted in a C-index decrease from 0.703 (in TCGA LUAD) to 0.621 (in the NCI study), an overall reduction of 11.66%, which may reflect the discrepancy between the study populations (the NCI study included early stage patients) or the evaluation criteria of clinical variables between the two studies. The combination of mRNA and clinical variables led to a lower C-index as well (0.591 in the NCI study and 0.698 in TCGA LUAD). This suggests that the trained kernel machine learning model based on mRNA may be more reliably applied to other studies than using clinical variables in certain conditions.

### Prognostic powers of protein expression

To investigate the prognostic value of protein expression, we analyzed the datasets based on Proteomics Reverse Phase Protein Array (RPPA) platform. Since the number of subjects available in a given cancer type is much smaller than those for other omic platforms, we examined the RPPA data separately. First, we investigated whether aggregating protein levels by kernel machine learning method would improve prognostic prediction, compared with selecting protein biomarkers by penalized regression (Lasso) methods, across cancer types. Consistent with other omic profiles, the C-indices by kernel machine learning method were significantly higher that the C-indices based on the Lasso model (Supplementary Figure 4), either including protein levels only (P = 0.025 by one-sided paired Wilcoxon signed rank test with continuity correction) or combining protein levels with clinical variables (P = 0.012). This indicates that prognosis effects of protein biomarker likely follow a continuous spectrum (similar to those observed for other platforms) and that aggregating prognosis effects of all protein biomarkers may be more effective. Then, we compared the prognostic value of protein levels by kernel machine learning method with that by mRNA across cancer types. As expected, they are highly correlated for omic profile only (spearman correlation rho = 0.62, P = 0.024) and for the combination with clinical variable (rho = 0.94, P < 0.001). Indeed, C-indices by mRNA and by protein levels were very similar (Supplementary Figure 5) both for omic profile only (P = 0.576 by two-sided paired Wilcoxon signed rank test with continuity correction) and for combining protein levels with clinical variables (P = 1.000). It suggests similar prognostic values of mRNA and protein profiles.

### Impact of the number of subjects or genes

The performance of prognostic prediction depends on the study sample size and the number of biomarkers involved. The proposed kernel machine learning method is no exception. We chose the BRCA mRNA-seq data set (which had the largest sample size of the 14 TCGA cancer types we analyzed) as a working example to examine how the C-index varies depending on the sample size and the number of mRNA transcripts used to build the kernel method through down-sampling analysis, which repeatedly samples part of subjects or genes with gradually reducing size and evaluates the prognostic performance (Methods).

**Figure 6:**
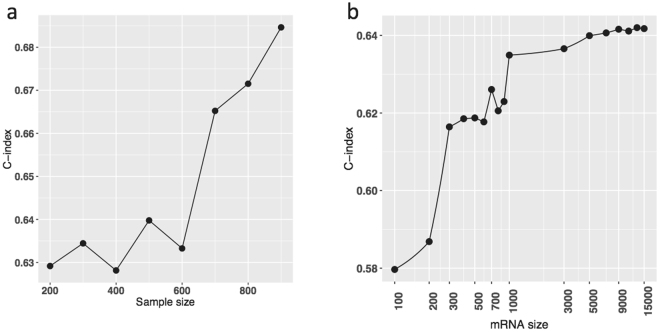
The dependence of prognostic assessment on the sample size and the number of biomarkers using a sub-sampling analysis of the BRCA mRNA-seq data set. (**a**) Scatter plot of C-indices by the increasing sample size. (**b**) Scatter plot of C-indices by the increasing number of mRNA transcripts.

We observed that the C-index steadily increases with increasing sample size and did not plateau even with over nine hundred subjects (Fig. 6a). This suggests that the current sample sizes are insufficient to fully achieve the optimal prognosis power of molecular data for BRCA and other cancer types, and an expanded patient cohort is needed for the kernel machine learning method and other statistical learning strategies to reach their full potential. Figure 6b shows the C-indices for various numbers of mRNA transcripts. Going from 1000 to 5000 markers improves the C-index substantially; a plateau is reached at around 5000 markers. Our down-sampling analysis implies that a large number of mRNA expression markers may contribute to refining the prognostic prediction.

## Discussion

Accurate prognostic assessment of cancer is of great value for patients, oncologists, and cancer researchers. Yet it remains challenging due to inter-tumor heterogeneity. The increasing popularity of multi-omic profiling of tumors raises the hope of improving prognostic prediction through the integration of clinical and omic biomarkers. We systematically evaluated the integration of clinical variables and omic profiles for survival prediction using a novel kernel machine learning method, which we applied to over three thousand tumor samples across fourteen cancer types from the TCGA. The kernel machine learning method built upon omic similarity matrices provides a comprehensive framework to incorporate multiple clinical variables and omic platforms simultaneously, yet it is intuitively simple and interpretable in which subjects with similar clinical variables and omic profiles have similar prognostic outcomes.

Genomic similarity matrices have been extensively used in genetic epidemiology studies^24,25^, but little has been explored in the context of survival prediction using high-dimensional genomic markers. Our study provides several unique contributions. First, most existing studies focus on genomic similarity matrices defined by SNPs only. In contrast, the proposed approach exploits information from multiple omic profiles. Secondly, the existing kernel methods have mostly been applied to study heritability of traits or disease risk^26,27^ and to analyze associations between a genetic similarity matrix and trait information in linear^28^ or logistic regression^29–32^ frameworks. In this study, we proposed a new kernel based prediction method for cancer prognosis. This framework allows us to incorporate clinical information and multiple omic profiles for the systematic assessment of prognostic performance across multiple cancer types in thousands of tumor samples.

The pan-cancer prognostic assessment confirmed that the difficulty of prognostic prediction varies considerably across cancer types, and that the utilities of prognostic profiles are unequal. Some cancer types, like LGG, demonstrate superior prognostic accuracy than others, based on clinical variables or molecular markers. Indeed, cancer type itself explains more than two thirds of C-index variability across cancer types. Among the different omics data types, mRNA expression most frequently provides the highest C-index for predicting patients’ survival outcome compared to the other molecular profiles in our analysis, suggesting that the resulting expression of mutated genes may be more important for patients’ survival than the underlying mutational patterns. DNA methylation and miRNA expression data also provide good prognostic values in several cancer types.

Cancer is extremely heterogeneous. We hypothesized that a small number of omic biomarkers would be unlikely to provide sufficient prognostic power. Instead, we proposed to aggregate numerous omic-wide prognostic biomarkers, using a genome-scale kernel machine learning method. Our approach consistently outperformed “condensed” signatures including the metagene signatures, immune signature, and lasso-regression derived prognostic signatures that rely only on the top-performing genes in the transcriptome. In addition, down-sampling of mRNA transcripts of BRCA suggested that thousands of transcripts are likely associated with prognosis. These observations were consistent with our hypothesis that a large number of biomarkers are involved in tumor prognosis in highly heterogeneous tumor types, and that each of the omic biomarkers has a small to moderate prognosis effect size following a distribution with long tail, and do not necessarily pass a genome-wide significance threshold. The omics-wide architecture of tumor prognosis we observed is different from the ones focusing on a relatively small number of omic biomarkers with relatively larger prognosis effect.

The long-tailed distribution of effect size we proposed for tumor prognosis is not unique but has been also observed in other biomedical research areas. For risk prediction in GWAS^33,34^, it has been reported that hundreds of variants with gradually decreased effect sizes are associated with complex polygenic traits, such as height^35,36^. In discovery and saturate analysis of cancer genes, it suggested that in addition to over two hundred known cancer genes, larger sample size will discover many more with lower mutation frequency^37^. Such a long-tailed distribution possibly reflects the complex mechanisms underlying the tumor prognosis, genetic architecture of complex traits, and nature selection of somatic mutation respectively.

A number of limitations of our prognosis analysis based on TCGA data warrant more discussion. First, the samples and clinical records of TCGA were collected retrospectively, and tended to over represent cases with fresh-frozen specimens of high quality, and large tumor size in late-stage patients. Hence, the kernel machine learning model trained on the TCGA samples may not be directly applicable to the general population. Second, the sample size of a given cancer type is limited, which may lead to unsaturated prognostic power and overfitting. Further studies with larger size, patients more representative of the general population, and with improved clinical records are necessary to further delineate the omic architecture of prognosis and achieve the full prognostic power of the kernel machine learning method, which should be evaluated in completely independent studies before being applied to clinical practice. Third, the clinical follow up is insufficient in the TCGA study cohorts for some cancer types, including for BRCA and prostate adenocarcinoma (PRAD). Combined with small sample size, this may contribute to the lack of significant increase in prediction performance for the integrated analysis. Fourth, most of subjects in TCGA received the standard treatment, such as surgery, chemotherapy and/or radiation therapy. New studies to evaluate the prognostic utility of omic profiling for newly developed targeted therapies and immunotherapies would be desireable. Finally, although six omic profiles have been examined, additional data types could further improve the precision of predicting clinical outcomes, including measures of intra-tumor heterogeneity, imaging, proteomics, and immunological factors. Our framework can be extended to accommodate these additional data types.

In conclusion, our work evaluates the prognostic value of multi-omic profiling integrated with clinical factors in thousands of samples across fourteen cancer types and proposes an omics-wide architecture of tumor prognosis. If confirmed in future studies, it suggests that genome-scale profiling platforms, instead of gene panels, should be preferred for future prognostic assessment and that the research focus should be shifted from molecular biomarker selection to large scale omic biomarker aggregation in the era of precision oncology.

## Methods

### Overview of the multi-omic kernel machine learning method and alternative approaches

We evaluated the prognostic value of six omic profiles and their combinations across cancer types by a multi-omic kernel machine learning method, which includes creating omic similarity matrices using kernels, kernel integration and survival prediction using a Cox kernel machine regression framework, and prognostic performance evaluation.

In addition to the multi-omic kernel machine learning method, we also considered several alternative approaches, including a conventional method considering clinical variables only, a sparse model involving variable selection^16^, and knowledge-driven models based on pre-defined metagenes^15^ or immune cell infiltration score^6^, both of which have demonstrated strong prognostic associations in several cancer types. For BRCA, we also applied the Cox model using the PAM50^21^ classification and the MammaPrint gene signature^22^. To obtain unbiased performance evaluation, we used cross-validation, fitting model in training datasets and evaluating the prognostic performance in validation datasets using the C-index^38^. The C-index, as a generalization of area under the receiver operating characteristics curve, is a widely used measure for model assessment in survival analysis that evaluates the proportion of subjects with both longer observed survival time and higher predicted probability of survival (i.e. the proportion of subjects correctly ranked for overall survival). The larger the C-index, the better the prognostic performance is, with a value of one indicating a perfect prognosis prediction and a value of 0.5 indicating a random prediction.

### Description of the datasets

We analyzed 3,382 samples across 14 TCGA cancer types. Rare cancer types were not included due to sample size limitations. Cancer types (e.g., prostate cancer) with very few events (death) were also excluded. Six different molecular profiles were used, including germline variants (SNP6.0 array), somatic point mutation (whole-exome sequencing), DNA copy number (SNP6.0 array), DNA methylation (Illumina Human Methylation 450 K array), mRNA expression (mRNA sequencing), and miRNA expression (miRNA sequencing, not included for four cancer types with very limited data on miRNA). Patient samples with all data types available were included in the analysis. Principal component analysis was used to examine and visualize potential batch effects. Batch effects were identified in four methylation data sets (BRCA, LUSC, UCEC, and KIRC). The ComBat algorithm^39^ implemented in the SVA package^40^ was used to adjust for batch effects. The TCGA datasets were obtained from the TCGA data portal (now the Genomic Data Commons) and the Broad Institute’s Firehose pipeline. Sample size, patient demographics, distribution of tumor stage and overall survival statistics are summarized in Supplementary Table 1. The clinical variables for each cancer type include age, stage, and additional well-known prognostic factors, such as Lauren classification in STAD. The number of biomarkers for each platform is listed in Supplementary Table 2.

### TCGA dataset compilation

Each individual data type was pre-processed using the following procedure. Copy number alteration data were derived from the segmented data using the Circular Binary Segmentation algorithm^41^, and further reduced to a set of non-redundant regions as described in Mo *et al*.^42^. For the methylation data (Illumina Infinium 450k arrays), a beta-mixture quantile normalization^43^ was applied to normalize the beta-value. Methylation probes with >20% or more missing data and those corresponding to SNP and autosomal chromosomes were removed. RNAseq version 2 was used. MapSplice^44^ was used for sequence alignment and RSEM^13^ for the quantitation of gene expression. For mRNA and miRNA sequence data, lowly-expressed genes were excluded based on median-normalized counts.

### Omic similarity matrix as kernel

Assuming there are *M* kinds of omic profiles. For the $m\,$th omic profile, we collected *p*_*m*_ omic biomarkers for *n* subjects which were organized into an *n* × *p*_*m*_ matrix **Z**_*m*_. Denote ***Z*** **′**_*m*_ as the transpose of **Z**_*m*_ and **Z**_*mj*_ as its *j*th column. For mRNA, miRNA, methylation, and copy number profiles, **Z**_*mj*_ is in the continuous scale and normalized with mean zero and variance one; for somatic mutation, **Z**_*mj*_ was recorded as binary values with zero for observing no somatic mutation at the *j* th gene and one otherwise. The corresponding linear kernel, an *n* × *n* matrix, was defined as

1 $${K}_{m}=\frac{({Z}_{m}Z{\text{'}}_{m})}{{p}_{m}}.$$

Kernel alignment^18^ measures the similarity between two kernels, namely *K*_1_ and *K*_2_, defined as

2 $$A({K}_{1},{K}_{2})=\frac{ < {K}_{1},{K}_{2} > }{\sqrt{ < {K}_{1},{K}_{1} >  < {K}_{2},{K}_{2} > }}$$

where $$ < {K}_{1},{K}_{2} > ={\sum }_{(i,j=1)}^{n}{K}_{1}(i,j)\,{K}_{2}(i,j)$$ is the inner product and $${K}_{1}(i,j)$$ is the matrix entry at the *i*th row and *j*th column. If $${K}_{1}(i,j)$$ and $${K}_{2}(i,j)$$ are identical, i.e. $${K}_{1}(i,j)$$ $$=$$ $${K}_{2}(i,j)$$, then $$A({k}_{1},{k}_{2})=1$$; if $${K}_{1}(i,j)$$ = $$-{K}_{2}(i,j)$$, then $$A({k}_{1},{k}_{2})=-1$$; and $$A({k}_{1},{k}_{2})=0$$, if $$ < {K}_{1},{K}_{2} > =0$$.

An omic similarity matrix could also be derived from the omic prognostic score, which is the weighted sum of all biomarkers in an omic profile with weights (alternatively called prognosis coefficients) following a univariate normal distribution with mean zero and a constant variance. Unlike the other prognosis indices commonly used with a handful of biomarkers, the omic prognostic score involves tens of thousands of biomarkers whose prognosis coefficients are treated as random and concentrate around zero. It can be easily shown that omic prognostic indices follow a multivariate normal distribution with mean zeros and covariate matrix proportional to the corresponding omic similarity matrix (details in the later section).

### Multi-omic kernel learning method for prognostic prediction

We propose a kernel-fusion Cox model as the multi-omic kernel learning method. Specifically, consider the Cox proportional hazards model,

3 $$\,{\lambda }_{i}(t)={\lambda }_{0}(t)\exp ({\eta }_{i}),$$

4 $$\,{\eta }_{i}={b}_{i}+{g}_{i},\,i=1,2,\cdots I$$

where ${{\rm{\lambda }}}_{{\rm{i}}}({\rm{t}})$ is the hazard function for the *i* th subject, ${{\rm{\lambda }}}_{0}({\rm{t}})$ the baseline hazard function, and ${{\rm{\eta }}}_{{\rm{i}}}$ the overall prognostic score. The prognostic score ${{\rm{\eta }}}_{{\rm{i}}}$ in turn is the sum of the clinical prognostic score ${{\rm{b}}}_{{\rm{i}}}$ and the omic prognostic score ${{\rm{g}}}_{{\rm{i}}}$. We specify ${{\rm{b}}}_{{\rm{i}}}=\sum \,_{{\rm{j}}}^{{\rm{n}}}{{\rm{\beta }}}_{{\rm{j}}}\,{{\rm{X}}}_{{\rm{ij}}}$ for ${\rm{n}}$ fixed-effect ${{\rm{X}}}_{{\rm{ij}}}$’s with fixed effect coefficient ${{\rm{\beta }}}_{{\rm{j}}}$’s. Denote ${\rm{\eta }}=({{\rm{\eta }}}_{1},{{\rm{\eta }}}_{2},\cdots \,{{\rm{\eta }}}_{{\rm{I}}})\text{'}$, ${\rm{b}}=({{\rm{b}}}_{1},{{\rm{b}}}_{2},\cdots \,{{\rm{b}}}_{{\rm{I}}})\text{'}$, and ${\rm{g}}=({{\rm{g}}}_{1},{{\rm{g}}}_{2},\cdots \,{{\rm{g}}}_{{\rm{I}}})\text{'}$ as the vectors of overall prognostic score, clinical prognostic score and omic prognostic score respectively. We assume ${\rm{g}}$ follows a multivariate normal distribution ${\boldsymbol{g}} \sim {\rm{N}}(0,{\rm{K}})$ with mean zero and variance-covariance matrix ${\rm{K}}$ as a fused kernel. Indeed, K $=\sum _{{\rm{m}}=1}^{{\rm{M}}}{{\rm{\sigma }}}_{{\rm{m}}}^{2}{{\rm{K}}}_{{\rm{m}}}$, a linear combination or fusion of multiple Omic similarity matrices ${{\rm{K}}}_{{\rm{m}}}$’s, corresponding to somatic mutation, mRNA, miRNA, methylation and copy number profiles. For germline variants, we focused on SNPs which were significant in the genome-wide association studies and created the PRS based on reported odds ratios. PRS was regarded as a fixed effect in the Cox model.

From the random effects perspective, we can show that ${\boldsymbol{g}}$ aggregates numerous omic biomarkers whose effects are treated as random effects coefficients shrunk toward zero. Indeed, we represent

5 $${\boldsymbol{\eta }}={\boldsymbol{b}}+{\boldsymbol{g}}={\boldsymbol{b}}+\sum _{{\boldsymbol{m}}=1}^{{\boldsymbol{M}}}{{\boldsymbol{g}}}_{{\boldsymbol{m}}}={\boldsymbol{b}}+\sum _{{\boldsymbol{m}}=1}^{{\boldsymbol{M}}}\sum _{{\boldsymbol{j}}\in {{\boldsymbol{R}}}_{{\boldsymbol{m}}}}{{\bf{Z}}}_{mj}{\alpha }_{{\boldsymbol{mj}}},$$

for which ${{\boldsymbol{g}}}_{{\boldsymbol{m}}}$ is the vector of omic prognostic score for the $m\,$th omic profile and the linear combination of omic biomarkers ${{\bf{Z}}}_{mj}$ with the random effects coefficient ${\alpha }_{mj}$. We assume ${\alpha }_{mj}$ follow normal distribution ${\alpha }_{mj} \sim N(0,\frac{{\sigma }_{m}^{2}}{{p}_{m}})$, it is straightforward to show that ${{\boldsymbol{g}}}_{{\boldsymbol{m}}} \sim {\boldsymbol{N}}(0,\,{\sigma }_{m}^{2}{K}_{m})$ and ${\boldsymbol{g}} \sim N(0,{\boldsymbol{K}})$.

### Model building, evaluation and comparison

We considered, evaluated, and compared several prognosis prediction methods through the Monte Carlo cross-validation. Given the method and cancer type, we randomly selected 80% of subjects as the training dataset and the remaining 20% of subjects as the validation dataset. For each training dataset, we fit (a) the Cox model with clinical variables only using R package “Survival”; (b) the kernel-fusion Cox models for one omic profile at a time and their pairwise combinations using R package “coxme”; (c) the kernel-fusion Cox models considering both clinical variables and omic profiles. To calculate the omic prognostic scores ${{\boldsymbol{g}}}_{V}$ for the subjects in the validation dataset, we first recorded subjects in the training dataset together and segmented ${\boldsymbol{K}}$ as

6 $${\boldsymbol{K}}=[\begin{array}{cc}{{\boldsymbol{K}}}_{VV} & {{\boldsymbol{K}}}_{VT}\\ {{\boldsymbol{K}}}_{TV} & {{\boldsymbol{K}}}_{TT}\end{array}],$$

where ${{\boldsymbol{K}}}_{VV}$ is the variance matrix for the validation dataset, ${{\boldsymbol{K}}}_{VT}$ is the covariance matrix between the validation dataset and the training dataset, and ${{\boldsymbol{K}}}_{TT}$ is the variance matrix for the validation dataset. We obtained the best linear unbiased predictor of omic prognostic scores ${\hat{{\rm{g}}}}_{{\rm{T}}}$ and restricted maximum likelihood estimators of ${\hat{\sigma }}_{m}^{2}$ from the fitted kernel-fusion Cox models, and the predicted omic prognostic scores was given as ${\hat{{\boldsymbol{g}}}}_{V}={{\boldsymbol{K}}}_{VT}{{\boldsymbol{K}}}_{TT}^{-1}{\hat{{\boldsymbol{g}}}}_{T}$. Similarly, the predicted clinical prognostic scores were calculated as ${\hat{{\boldsymbol{b}}}}_{V}=\sum _{j}^{n}{\hat{\beta }}_{j}\,{{\boldsymbol{X}}}_{Vj}$, for which ${\hat{\beta }}_{j}$ is the maximum likelihood estimator of ${\beta }_{j}$ for the fit the Cox model or the kernel-fusion Cox models and ${{\boldsymbol{X}}}_{Vj}$ is the vector of *j*th clinical variable for subjects in the validation dataset. Finally, the predicted overall prognostic scores are given as ${\hat{{\boldsymbol{\eta }}}}_{{\boldsymbol{V}}}=\,{\hat{{\boldsymbol{b}}}}_{V}+{\hat{{\boldsymbol{g}}}}_{V}$. Comparing the order of predicted overall prognostic scores with the order of death events time for all subject pairs in the training dataset, we could calculate a C-index using the R package ‘Coxph’ for one Monte Carlo cross-validation, which then was repeated 100 times and the average C-index was calculated and reported.

For mRNA profiles, we further considered (a) a Cox model with LASSO for the omic biomarker selection, the details of which was given by Yuan *et al.* ^16^; (b) a Cox model with predefined prognosis signatures: immune and metagenes. The calculated immune signatures as the immune cell immune infiltration scores were downloaded from http://bioinformatics.mdanderson.org/estimate/. The metagene signatures included CIN, MES and LYM attractor metagenes, whose levels were calculated as the average of the mRNA expression levels of ten top-ranked genes^15^. For breast cancer, we also applied the Cox model using PAM50^21^ and mammaPrint gene list^22^.

### Linear mixed model for comparing prognostic powers across cancer types

The prognostic powers of omic profiles, measured by C-indices, for a given cancer type were related. This relation was not surprising. The clinical variables and omic profiles were measured on the same subjects, and we have shown that the omic similarity matrices were well aligned. Hence, we applied linear mixed models to quantify and compare the contribution of clinical variables and omic profiles to prognostic powers across cancer types, while considering their between-cancer correlation. The linear mixed models were fit by the R package “lme4” and P-values were given by R package “lmerTest”.

We first quantified the contribution of cancer type itself to the variation of C-indices for clinical variables and omic profiles. Denote ${y}_{ij}$ as the C-index of the *j*th omic profile or clinical variables in the *i*th cancer type, the following model assumed that the variation of ${y}_{ij}$ originated from two resources, the cancer type itself and anything else, including clinical variables and omic profiles we chosen and unknown factors. Specifically,

7 $$\,{y}_{ij}={\beta }_{0}+{\delta }_{i}+{{\epsilon }}_{ij},\,i=1,2,\cdots I,\,j=1,2,\cdots {n}_{i},$$

where ${\beta }_{0}$ is the average C-index, ${\delta }_{i}\,\,$measures the contribution of cancer type and ${{\epsilon }}_{ij}$ anything else. We assumed ${\delta }_{i}\,\,$and ${{\epsilon }}_{ij}$ followed normal distributions as ${\delta }_{i} \sim N(0,\,{\sigma }_{b}^{2})$ and ${{\epsilon }}_{ij} \sim N(0,\,{\sigma }_{\varepsilon }^{2})$. The total variation of C-indices was ${\sigma }_{b}^{2}+{\sigma }_{\varepsilon }^{2}$ and the proportion of total variation due to the cancer type was $S=\frac{{\sigma }_{b}^{2}}{{\sigma }_{b}^{2}+{\sigma }_{\varepsilon }^{2}}$. We named it inter-profile heterogeneity. An inter-profile heterogeneity of one meant that C-indices by multiple clinical variables and omic profiles for a given cancer type were all the same and there was no inter-profile heterogeneity of C-indices. On the other extreme, an inter-profile heterogeneity of zero implied the C-idexes by multiple clinical variables and omic profiles for a given cancer type were unrelated, and that cancer type contributes none to the C-index. The inter-profile heterogeneity observed in real studies likely lies in between these two extremes.

A number of modified linear mixed models were applied to compare between clinical variables and omic profiles, and between omic profiles themselves. For example, we used linear mixed model ${y}_{ij}={\beta }_{0}+{\beta }_{1}{x}_{ij}+{\delta }_{i}+{{\epsilon }}_{ij}$ to compare the prognostic powers of clinical variables and omic profiles, for which ${x}_{ij}$ is an binary indicator, equaling to one if ${y}_{ij}$ was obtained based on clinical variables and zero otherwise; linear mixed model ${y}_{ij}={\beta }_{0}+\sum _{k=1}^{M}{\beta }_{k}{x}_{kij}+{\delta }_{i}+{{\epsilon }}_{ij}$ to compare the prognostic powers between somatic mutation, mRNA, miRNA, copy number, and methylation profiles, each of which were indicated by the corresponding binary indicator ${x}_{kij}$, one as using the *k*th profiles and zero otherwise; linear mixed model ${y}_{ij}={\beta }_{0}+{\beta }_{1}{z}_{ij}+{\delta }_{i}+{{\epsilon }}_{ij}$ to compare C-indices by two profiles versus by one profile; linear mixed model ${y}_{ij}={\beta }_{0}+\sum _{k=1}^{3}{\beta }_{k}{w}_{kij}+{\delta }_{i}+{{\epsilon }}_{ij}$ to compare C-indices by Lasso selection, metagene, or immune signatures, indicated by ${w}_{kij}$, with C-indices using all mRNA by kernel learning method.

### Down-sampling of subjects or genes

Among 16,598 mRNA transcripts, we randomly selected a subset of transcripts, built kernel, ran fivefold cross-validation for the kernel learning method, and calculated C-indices. The procedure was essentially the same as the one for the complete set of transcripts with one key difference: down-sampling of transcripts. The number of transcripts was gradually reduced until reaching one hundred. For a given number of transcripts, the down-sampling was conducted fifty times and the average C-index was calculated. Similarly, we carried out down-sampling of subjects, i.e. selecting a subset of subjects which gradually reduced until reaching two hundred. For selected subjects, we used all mRNA transcripts to build the kernel, carried out fivefold cross-validation for the kernel learning method, and obtained the C-index. This procedure was repeated fifty times for a given reduced sample size and the average C-index was reported.

### Data availability of data and materials

The TCGA datasets were obtained from the TCGA data portal (now the Genomic Data Commons) and the Broad Institute’s Firehose pipeline.

## Electronic supplementary material

Supplementary Information

## References

[1] Vargas AJ, Harris CC (2016). Biomarker development in the precision medicine era: lung cancer as a case study. Nature Reviews Cancer.

[2] Ludwig JA, Weinstein JN (2005). Biomarkers in cancer staging, prognosis and treatment selection. Nat Rev Cancer.

[3] Lawrence MS (2013). Mutational heterogeneity in cancer and the search for new cancer-associated genes. Nature.

[4] Alexandrov LB (2013). Signatures of mutational processes in human cancer. Nature.

[5] Witte T, Plass C, Gerhauser C (2014). Pan-cancer patterns of DNA methylation. Genome Med.

[6] Yoshihara K (2013). Inferring tumour purity and stromal and immune cell admixture from expression data. Nature communications.

[7] Jacobsen A (2013). Analysis of microRNA-target interactions across diverse cancer types. Nature structural & molecular biology.

[8] Gentles AJ (2015). The prognostic landscape of genes and infiltrating immune cells across human cancers. Nat Med.

[9] Akbani R (2014). A pan-cancer proteomic perspective on The Cancer Genome Atlas. Nature communications.

[10] National Cancer Institute Cancer Moonshot Blue Ribbon Panel Report. https://www.cancer.gov/research/key-initiatives/moonshot-cancer-initiative/blue-ribbon-panel (2016).

[11] van ‘t Veer LJ (2002). Gene expression profiling predicts clinical outcome of breast cancer. Nature.

[12] Beer DG (2002). Gene-expression profiles predict survival of patients with lung adenocarcinoma. Nat Med.

[13] Mankoo PK, Shen R, Schultz N, Levine DA, Sander C (2011). Time to recurrence and survival in serous ovarian tumors predicted from integrated genomic profiles. PLoS One.

[14] Kim H (2010). Integrative genome analysis reveals an oncomir/oncogene cluster regulating glioblastoma survivorship. Proc Natl Acad Sci USA.

[15] Cheng, W. Y., Ou Yang, T. H. & Anastassiou, D. Development of a prognostic model for breast cancer survival in an open challenge environment. *Sci Transl Me*d 5, 181ra150 (2013). 10.1126/scitranslmed.3005974 23596202

[16] Yuan Y (2014). Assessing the clinical utility of cancer genomic and proteomic data across tumor types. Nat Biotechnol.

[17] Ein-Dor L, Zuk O, Domany E (2006). Thousands of samples are needed to generate a robust gene list for predicting outcome in cancer. Proceedings of the National Academy of Sciences of the United States of America.

[18] Cristianini, N., Shawe-Taylor, J., Elisseeff, A. & Kandola, J. On kernel-target alignment. *Advances in Neural Information Processing Systems* 14, *Vols 1 and 2* **14**, 367–373 (2002).

[19] Hirschhorn JN, Daly MJ (2005). Genome-wide association studies for common diseases and complex traits. Nat Rev Genet.

[20] McCarthy MI (2008). Genome-wide association studies for complex traits: consensus, uncertainty and challenges. Nat Rev Genet.

[21] Parker JS (2009). Supervised risk predictor of breast cancer based on intrinsic subtypes. J Clin Oncol.

[22] van de Vijver MJ (2002). A gene-expression signature as a predictor of survival in breast cancer. New England Journal of Medicine.

[23] Shi J (2016). Somatic Genomics and Clinical Features of Lung Adenocarcinoma: A Retrospective Study. PLoS Med.

[24] Schaid DJ (2010). Genomic similarity and kernel methods I: advancements by building on mathematical and statistical foundations. Hum Hered.

[25] Schaid DJ (2010). Genomic Similarity and Kernel Methods II: Methods for Genomic Information. Human Heredity.

[26] Sampson JN (2015). Analysis of Heritability and Shared Heritability Based on Genome-Wide Association Studies for Thirteen Cancer Types. J Natl Cancer Inst.

[27] Yang J (2010). Common SNPs explain a large proportion of the heritability for human height. Nat Genet.

[28] Liu D, Lin X, Ghosh D (2007). Semiparametric regression of multidimensional genetic pathway data: least-squares kernel machines and linear mixed models. Biometrics.

[29] Liu D, Ghosh D, Lin X (2008). Estimation and testing for the effect of a genetic pathway on a disease outcome using logistic kernel machine regression via logistic mixed models. BMC Bioinformatics.

[30] Cai T, Lin X, Carroll RJ (2012). Identifying genetic marker sets associated with phenotypes via an efficient adaptive score test. Biostatistics.

[31] Wu MC (2011). Rare-variant association testing for sequencing data with the sequence kernel association test. Am J Hum Genet.

[32] Lee S, Abecasis GR, Boehnke M, Lin XH (2014). Rare-Variant Association Analysis: Study Designs and Statistical Tests. American Journal of Human Genetics.

[33] Park JH (2010). Estimation of effect size distribution from genome-wide association studies and implications for future discoveries. Nat Genet.

[34] Chatterjee N (2013). Projecting the performance of risk prediction based on polygenic analyses of genome-wide association studies. Nat Genet.

[35] Wood AR (2014). Defining the role of common variation in the genomic and biological architecture of adult human height. Nat Genet.

[36] Marouli E (2017). Rare and low-frequency coding variants alter human adult height. Nature.

[37] Lawrence MS (2014). Discovery and saturation analysis of cancer genes across 21 tumour types. Nature.

[38] Harrell FE, Lee KL, Mark DB (1996). Multivariable prognostic models: issues in developing models, evaluating assumptions and adequacy, and measuring and reducing errors. Statistics in medicine.

[39] Johnson WE, Li C, Rabinovic A (2007). Adjusting batch effects in microarray expression data using empirical Bayes methods. Biostatistics.

[40] Leek JT, Storey JD (2007). Capturing heterogeneity in gene expression studies by surrogate variable analysis. PLoS Genet.

[41] Olshen AB, Venkatraman ES, Lucito R, Wigler M (2004). Circular binary segmentation for the analysis of array-based DNA copy number data. Biostatistics.

[42] Mo Q (2013). Pattern discovery and cancer gene identification in integrated cancer genomic data. Proc Natl Acad Sci USA.

[43] Pidsley R (2013). A data-driven approach to preprocessing Illumina 450K methylation array data. BMC Genomics.

[44] Wang K (2010). MapSplice: accurate mapping of RNA-seq reads for splice junction discovery. Nucleic Acids Res.

