# Supplementary material for: Integrating Clinical and Multiple Omics Data for Prognostic Assessment across Human Cancers
Source: Sci Rep. 2017 Dec 5;7:16954. doi: 10.1038/s41598-017-17031-8 (PMC5717223; doi:10.1038/s41598-017-17031-8)
Supplement: Supplementary file 1 — Supplementary Information [file 41598_2017_17031_MOESM1_ESM.pdf]

Integrating Clinical and Multiple Omics Data for Prognostic Assessment across Human Cancers

Bin Zhu[1,*], Nan Song[2,*], Ronglai Shen[3,*], Arshi Arora[3], Mitchell J. Machiela[1], Lei Song[1], Maria Teresa Landi[1], Debashis Ghosh[4], Nilanjan Chatterjee[5,6], Veera Baladandayuthapani[7] and Hongyu Zhao[8]

Affiliations:
1. Division of Cancer Epidemiology and Genetics, National Cancer Institute, National Institute of Health, Bethesda, MD 20892, USA
2. NSABP Foundation, Pittsburgh, PA 15212, USA
3. Department of Epidemiology and Biostatistics, Memorial Sloan-Kettering Cancer Center, New York, NY 10021, USA
4. Department of Biostatistics and Informatics, University of Colorado Denver, Aurora, CO 80045, USA
5. Department of Biostatistics, Bloomberg School of Public Health, Johns Hopkins University, Baltimore, MD 21205, USA
6. Department of Oncology, School of Medicine, Johns Hopkins University, Baltimore, MD 21205, USA
7. Department of Biostatistics, The University of Texas M. D. Anderson Cancer Center, Houston, TX 77230, USA
8. Department of Biostatistics, Yale School of Public Health, New Haven, CT 06520, USA
* Authors contributed equally

Running title: Integrative cancer prognostic assessment

Correspondences to:

Bin Zhu, Ph.D.
Principal Investigator
Biostatistics Branch
Division of Cancer Epidemiology and Genetics
National Cancer Institute
9609 Medical Center Drive, Room 7E618
Bethesda, MD 20892
bin.zhu@nih.gov

Conflict of interest: The authors have nothing to disclose

Supplementary tables and figures:

Table S1. Summary of patient demographic and clinical characteristics.

Table S2. The number of biomarkers in each omic platform across cancer types.

Figure S1. Pairwise kernel alignment among the five omic platforms (somatic mutation, copy number, methylation, mRNA and miRNA) in individual cancer types.

Figure S2. Pairwise Spearman correlation pattern in C-index between omic platforms.

Figure S3. Radar plot of C-index for individual and combination of omic profiles in individual cancer types alone (red lines), or in combination with clinical variables (light blue lines). The C-index for clinical variables alone are plotted in dotted dark blue lines.

Figure S4. Boxplots of C-indices of protein expression for various across cancer types by kernel machine learning method and Lasso respectively.

Figure S5. Boxplots of C-indices of protein expression and mRNA across cancer types

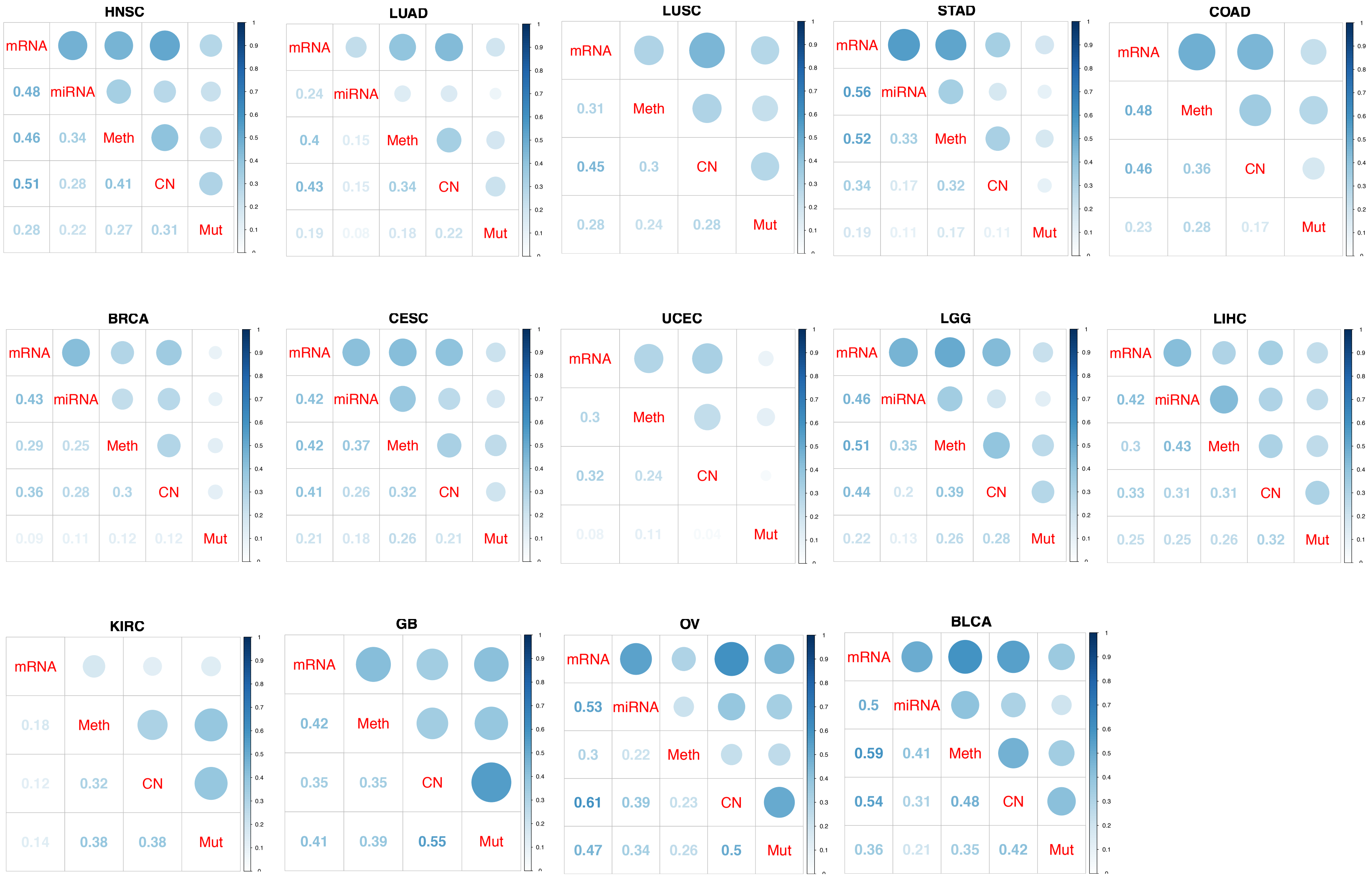

Supplementary Figure 1: Kernel alignment for each cancer type

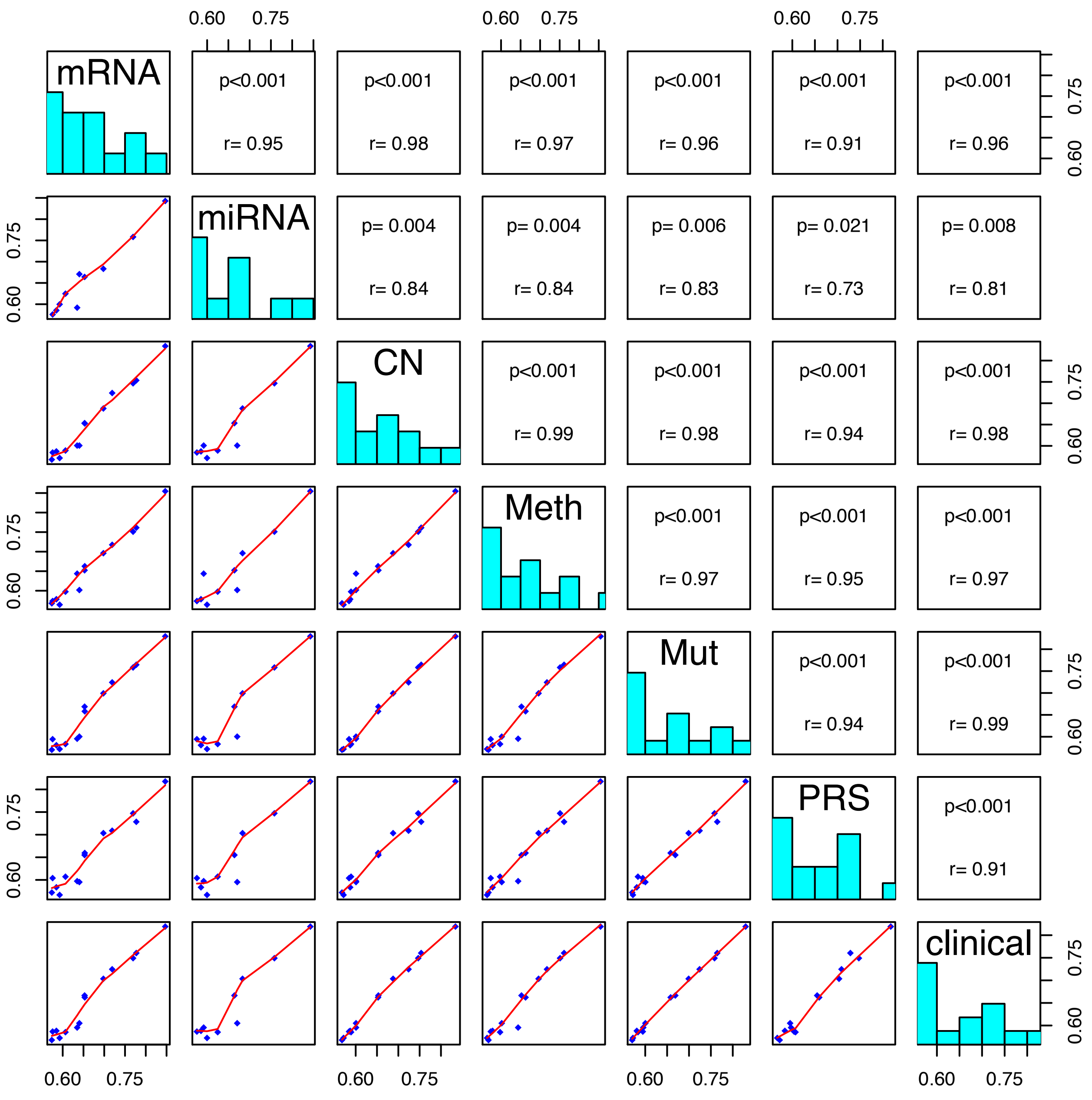

Supplementary Figure 2: Correlation plot including clinical factors

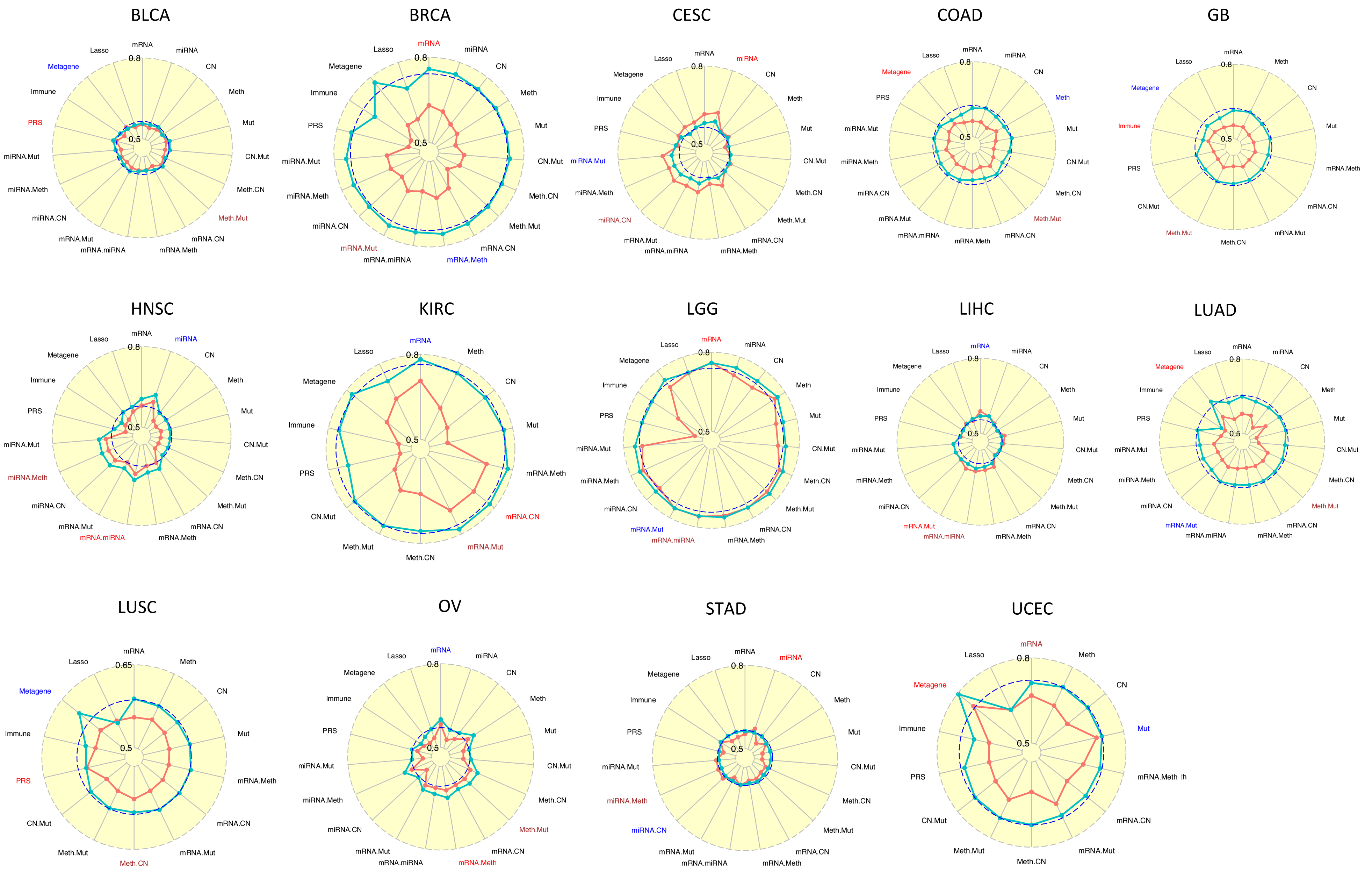

Supplementary Figure 3: Radar plots

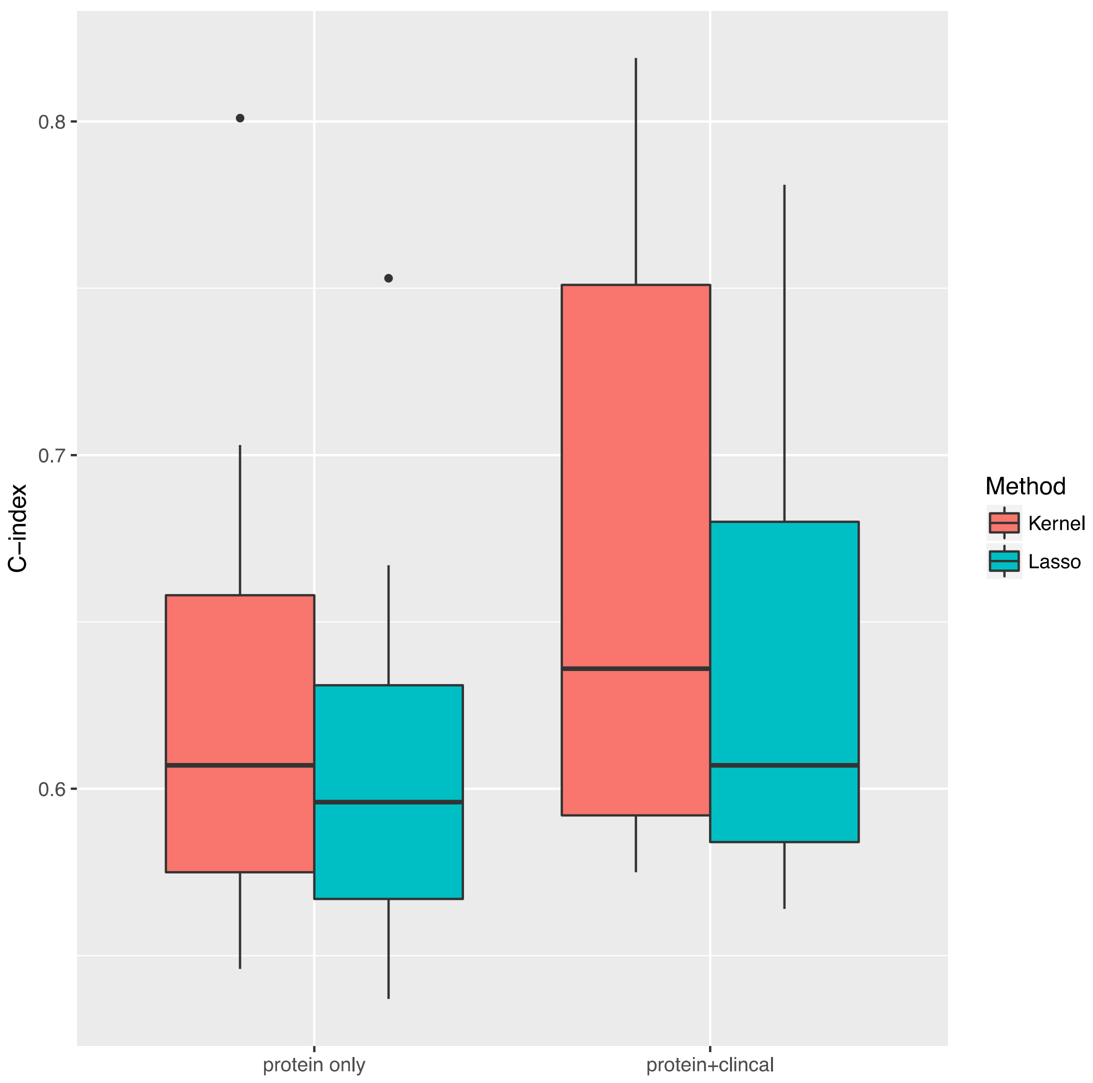

Supplementary Figure 4. Boxplots of C-indices of protein expression.

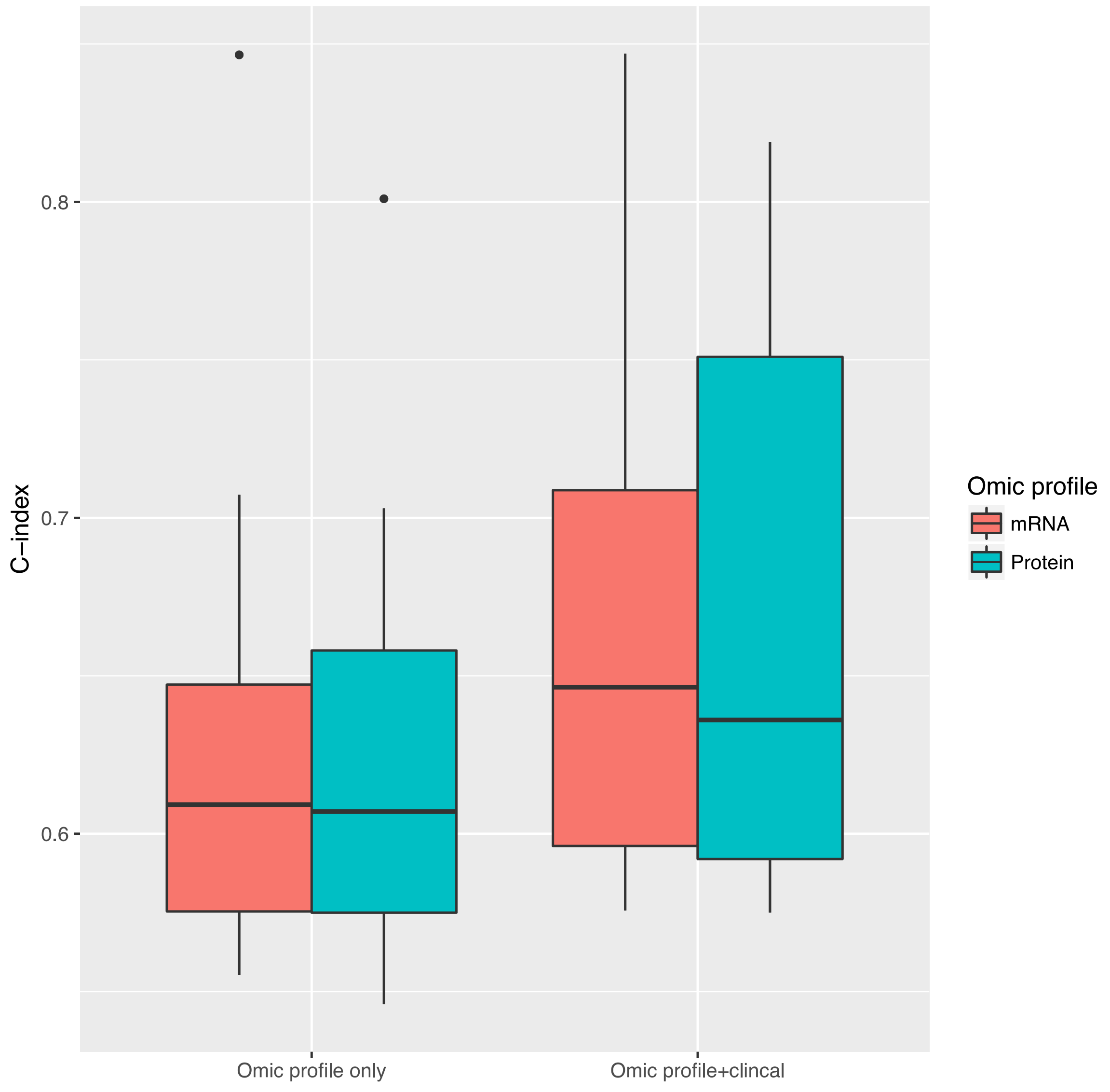

Supplementary Figure 5. Boxplots of C-indices of protein expression and mRNA

Supplemntary Table 1

Cancer	Total	Clinical information	Level	Counts
BLCA	124	age		mean: 67.98; sd: 10.48
		gender	Male	93
			Female	31
		stage	stage12	88
			stage34	36
		survival	0	64
			1	60
BRCA	623	age		mean: 58.45; sd:13.06
		stage	stage12	147
			stage34	476
		survival	0	529
			1	94
CESC	186	age		mean: 46.95; sd: 12.87
		stage	stage12	149
			stage34	37
		survival	0	145
			1	41
COAD	182	age		mean:65; sd:13.35
		stage	stage12	105
			stage34	77
		gender	Female	81
			Male	101
		survival	0	143
			1	39
GB (4)	66	age		mean:73.64; sd: 16.51
		gender	female	19
			male	47
		karnofsky_performance_score		mean:61.81; sd: 14.18
		survival	0	0
			1	66
HNSC	210	age		mean:61.72; sd:12.37
		stage	stage12	56
				154
		neoplasm_histologic_grade	G1	20
			G2	131
			G3,G4,GX	59
		survival	0	99
			1	111

KIRC4	407	age		mean:60.85;sd:11.98
		stage	stage1	232
			stage2	108
			stage34	67
		gender	female	140
			male	267
		grade	1	341
			3	66
		survival	0	273
			1	134
LUSC4	174	age		mean:67.62; sd: 8.37
		gender	female	47
			male	127
		stage	stage1	99
			stage2	37
			stage34	38
		survival	0	89
			1	85
LGG	277	age		mean:42.85, sd:13.39
		gender	female	123
			male	154
		grade	grade2	136
			grade3	141
		histology type	Astrocytoma	95
			Oligoastrocytoma	74
			Oligodendroglioma	108
		survival	0	219
			1	58
LIHC	163	age		mean:59.42; sd:13.83
		gender	female	54
			male	109
		grade	G1	27
			G2	83
			G3	53
		stage	stage12	108
			stage34	55
		survival	0	94
			1	69
LUAD	378	age		mean:65.52; sd:9.78
		gender	female	203
			male	175
		stage	stage1	205

			stage2	90
			stage34	83
		survival	0	94
			1	69
OV	144	age		mean:60.40, sd: 10.65
		stage	stage12	9
			stage34	135
		grade	G2	12
			G3	132
		TUMORRESIDUALDISEAS	1-10 mm	78
			11-20 mm	11
			>20mm	24
			No Macroscopic disease	31
		survival	0	52
			1	92
STAD	215	age		mean:65.45; sd: 10.34
		gender	female	80
			male	135
		stage	stage12	111
			stage34	104
		Lauren.Class	diffuse	55
			Intestinal	141
			Mixed	19
		survival	0	140
			1	75
UCEC4	233	age		mean:63.22; sd:10.91
		stage	stage12	181
			stage34	52
		Tumor Grade	grade 1	70
			grade 2	72
			grade 3	91
		histology	serous	42
			endometrioid+Mixed	191
		survival	0	210
			1	23

Supplementary Table 2

Cancer	N	Meth (Methylation 450K)	mRNA (mRNA-seq)	CN (SNP6)	Mut (whole-exome DNA-seq)	miRNA
BLCA	124	384200	16297	2855	13382	482
BRCA	623	22479	16958	9783	17689	833
CESC	186	383925	16284	8424	16453	492
COAD	182	22479	16359	6766	18060	443
GB	66	22474	12042	2821	9680	
HNSC	210	384167	16598	9761	14155	496
KIRC	407	23378	17118	1695	13769	
LGG	277	384208	16908	4717	5960	506
LIHC	163	385769	15687	969	11759	467
LUAD	378	384146	16647	9205	21056	386
LUSC	174	23523	16850	676	14872	
OV	144	22481	16882	2990	10060	389
STAD	215	383984	16654	1153	17172	464
UCEC	233	22488	16472	523	19649	

N: Number of samples included in the analysis (with all four data tupes available)

CN: number of unique copy number regions. The number of regions vary across cancer type depending on the complexity of copy number landscpe in each disease.

Meth: SNP and Sex vhromosome probes were removed. KNN imputation was performed to impute missing value. Porbes with greater than 20% missing were removed. Note that some of the disease types in ealier phase of TCGA including GBM, OV, KIRC, LUSC are 27K array data.

Mutation: list number of genes with at least one mutation observed in the cohort

mRNA: note RNA-seq data not available for GBM, Affy u133a array data was used for GBM
